# Lipid profiles and outcomes of patients with prior cancer and subsequent myocardial infarction or stroke

**DOI:** 10.1038/s41598-021-00666-z

**Published:** 2021-10-27

**Authors:** Chieh Yang Koo, Huili Zheng, Li Ling Tan, Ling-Li Foo, Raymond Seet, Jun-Hua Chong, Derek J. Hausenloy, Wee-Joo Chng, A. Mark Richards, Chi-Hang Lee, Mark Y. Chan

**Affiliations:** 1 Department of Cardiology, National University Heart Centre Singapore, Singapore, Singapore; 2 Cardiac Department, Yong Loo Lin School of Medicine, National University of Singapore, 1E, Kent Ridge Road, NUHS Tower Block, Level 9, Singapore, 119228 Singapore; 3 Health Promotion Board, National Registry of Diseases Office, Singapore, Singapore; 4 National Heart Centre Singapore, Singapore, Singapore; 5 Cardiovascular and Metabolic Disorders Program, Duke-National University of Singapore Medical School, Singapore, Singapore; 6 National Heart Research Institute Singapore, National Heart Centre, Singapore, Singapore; 7 The Hatter Cardiovascular Institute, University College London, London, UK; 8 Department of Haematology-Oncology, National University Cancer Institute of Singapore, Singapore, Singapore; 9 Christchurch Heart Institute, University of Otago, Dunedin, New Zealand

**Keywords:** Cardiology, Cancer, Risk factors

## Abstract

Patients with cancer are at increased risk of myocardial infarction (MI) and stroke. Guidelines do not address lipid profile targets for these patients. Within the lipid profiles, we hypothesized that patients with cancer develop MI or stroke at lower low density lipoprotein cholesterol (LDL-C) concentrations than patients without cancer and suffer worse outcomes. We linked nationwide longitudinal MI, stroke and cancer registries from years 2007–2017. We identified 42,148 eligible patients with MI (2421 prior cancer; 39,727 no cancer) and 43,888 eligible patients with stroke (3152 prior cancer; 40,738 no cancer). Median LDL-C concentration was lower in the prior cancer group than the no cancer group at incident MI [2.43 versus 3.10 mmol/L, adjusted ratio 0.87 (95% CI 0.85–0.89)] and stroke [2.81 versus 3.22 mmol/L, adjusted ratio 0.93, 95% CI 0.91–0.95)]. Similarly, median triglyceride and total cholesterol concentrations were lower in the prior cancer group, with no difference in high density lipoprotein cholesterol. Prior cancer was associated with higher post-MI mortality [adjusted hazard ratio (HR) 1.48, 95% CI 1.37–1.59] and post-stroke mortality (adjusted HR 1.95, 95% CI 1.52–2.52). Despite lower LDL-C concentrations, patients with prior cancer had worse post-MI and stroke mortality than patients without cancer.

## Introduction

Due to improvements in early detection and treatment, patients with cancer are living longer^1,2^. Multiple reports have confirmed an elevated risk of myocardial infarction (MI) and stroke among patients with prior cancer^3–7^. Possible reasons for this increased susceptibility to cardiovascular disease among cancer survivors include a persistent pro-inflammatory state and vascular toxicity from cancer treatments^8,9^.

Abnormal lipid profiles have been associated with increased risk of MI and stroke. Specifically, circulating low density lipoprotein cholesterol (LDL-C) has been identified as a major risk factor for MI and stroke^10^. Despite clear guidelines on the treatment and LDL-C targets for the general community, there are no specific targets for patients with prior cancer^10–13^. This is despite LDL-C being a common risk factor for both cardiovascular disease and cancer^14^. We hypothesized that patients with prior cancer may develop incident MI or stroke at lower LDL-C thresholds than patients without prior cancer and incur worse outcomes following these adverse events.

We linked data from nationwide registries to examine the circulating lipid concentrations of patients who presented with MI or stroke, and compared those with prior cancer to those without. Our objectives were first, to compare lipid profiles including high density lipoprotein cholesterol (HDL-C), triglyceride, total cholesterol and most importantly LDL-C concentrations at the time of incident MI or stroke diagnosis between patients with and without prior cancer; second, to determine if the relationship in LDL-C concentrations between prior cancer and no cancer at the point of incident MI would differ from the relationship in LDL-C concentrations between prior cancer and no cancer at the point of incident stroke; third, to determine if patients with prior cancer had worse post-MI and post-stroke outcomes than patients without cancer, and fourth, to assess the impact of preventative treatment with prior lipid lowering therapy (LLT) in patients with prior cancer.

## Methods

### Study design and participants

In this population-based cohort study, we combined data from three national registries managed by the National Registry of Diseases Office of Singapore^15^. Notification of selected diseases is mandatory under the National Registry of Diseases Act, which helps to ensure comprehensive coverage of the cases diagnosed or treated in Singapore. The Singapore Myocardial Infarction Registry comprises retrospectively collected records from all public and private hospitals of patients presenting with MI since 2007. Information such as to demographics, clinical presentation, inpatient laboratory and echocardiographic values, and pharmacotherapy prior to and at discharge were collected by trained coordinators^16^.

The Singapore Stroke Registry similarly comprises retrospectively collected records from all public hospitals of all patients presenting with stroke since 2005. This database captures patient demographics, clinical presentation, comorbidities, laboratory test results, medications and treatment details as previously described^17^. While the Singapore Myocardial Infarction Registry captures data on LLT and treatment for hypertension and diabetes mellitus initiated prior to incident MI, the Singapore Stroke Registry only captures data on the history of hyperlipidaemia, hypertension and diabetes mellitus without data on prior LLT and other respective treatment.

The Cancer Registry comprises retrospectively collected records across all public and private hospitals of all patients diagnosed with cancer since 1968. This database captures patient demographics, diagnostic details and treatment within the first six months from diagnosis as previously described^18^.

Data linkage between the Singapore Myocardial Infarction Registry and Singapore Stroke Registry with the Cancer Registry was performed using patients’ unique National Registration Identity Card number to determine if there was a prior diagnosis of cancer for patients presenting with MI or stroke in January 1, 2007 to 31 December, 2017. The data was then further matched with data from the Registry of Birth and Death to obtain the survival status of all patients. The reporting of deaths is mandatory by law in Singapore. Patients with incomplete lipid profile data, and those who developed cancer after incident MI or stroke were excluded.

The primary outcome of interest was LDL-C concentration at presentation of MI or stroke. Secondary outcomes of interest included measures of total cholesterol, HDL-C, triglyceride concentrations, and post-MI or post-stroke mortality. The MI and stroke patients were divided into two groups for analysis: one with a prior diagnosis of cancer and the other without a prior diagnosis of cancer (control group).

Missing data were excluded from the analyses through case deletion without imputation to maintain data in its original form. All analyses were done using STATA SE version 13 and based on de-identified data. This study was approved by the local institutional review board (Domain Specific Review Board-C, National Healthcare Group, 2013/00442) with waiver of consent approved for studies with public health importance using de-identified registries’ data. All methods were carried out in accordance with relevant guidelines and regulations.

### Statistical analysis

The characteristics and lipid profiles of patients in the prior cancer group and no cancer group were compared using Chi-square test for categorical variables and Wilcoxon rank-sum test for numeric variables as most of the numeric variables were not normally distributed.

To examine the relationship between LDL-C concentration and MI in the prior cancer and no cancer groups, the proportion of MI patients at each 1 mmol/L interval of LDL-C concentration was calculated and plotted for patients in these two groups. Similar approach was done for the stroke cohort, and for MI patients with history of hyperlipidemia to see if the relationship differed between those with or without prior LLT.

To determine if the lipid concentration at MI was affected by having a prior history of cancer, linear regression was done using natural logarithmic lipid concentration as the outcome, with prior cancer/no cancer group as the independent variable, adjusting for potential confounders available in our study (age at MI, sex, body mass index, prior treatment for hypertension, prior treatment for diabetes mellitus, prior treatment with LLT) in the multivariable models. Within the MI cohort, we performed similar linear regression in the subgroups of patients stratified by whether there was prior treatment with LLT. Similarly, within the stroke cohort, we adjusted for age at stroke, sex, history of hypertension, history of diabetes mellitus, history of hyperlipidaemia, and history or newly diagnosed atrial fibrillation or flutter in the multivariable linear regression models. As the lipid concentrations were not normally distributed, they were logarithmically transformed before analyzing as geometric means (instead of arithmetic means without any transformation) in the linear regression models.

Among patients with a cancer diagnosis preceding their first MI or stroke in the study period, the LDL-C concentration at incident MI or stroke was compared across subgroups of patients with various cancer characteristics at the point of cancer diagnosis using Kruskal–Wallis rank test and Chi-square test for numeric and categorical variables respectively. As the lipid concentrations were not normally distributed, medians (instead of means) and non-parametric (instead of parametric) tests were used for comparison.

To determine if having a prior history of cancer conferred a greater risk of all-cause death, cox regression was done using time from MI to death or censor (whichever earlier) as the outcome, with prior cancer/no cancer as the independent variable, adjusting for potential confounders (age at MI, sex, body mass index, smoking, prior treatment for hypertension, prior treatment for diabetes mellitus, LDL-C, left ventricular ejection fraction, ST-segment elevation MI, cardiac arrest, heart failure, and revascularisation) in the multivariable models. Similarly, within the stroke cohort, we adjusted for age at stroke, sex, smoking, history of hypertension, history of diabetes mellitus, history of hyperlipidaemia, history of transient ischaemic attack or stroke, history of ischaemic heart disease, history or newly diagnosed atrial fibrillation or flutter, LDL-C, and baseline National Institutes of Health Stroke Scale (NIHSS) score. Specifically for MI patients with history of hyperlipidemia, to further determine the impact of prior LLT on post-MI mortality, cox regression was done using time from MI to death or censor (whichever earlier) as the outcome, with prior LLT/no LLT as the independent variable, adjusting for the same potential confounders as mentioned above.

## Results

### Baseline demographics

**Figure 1:**
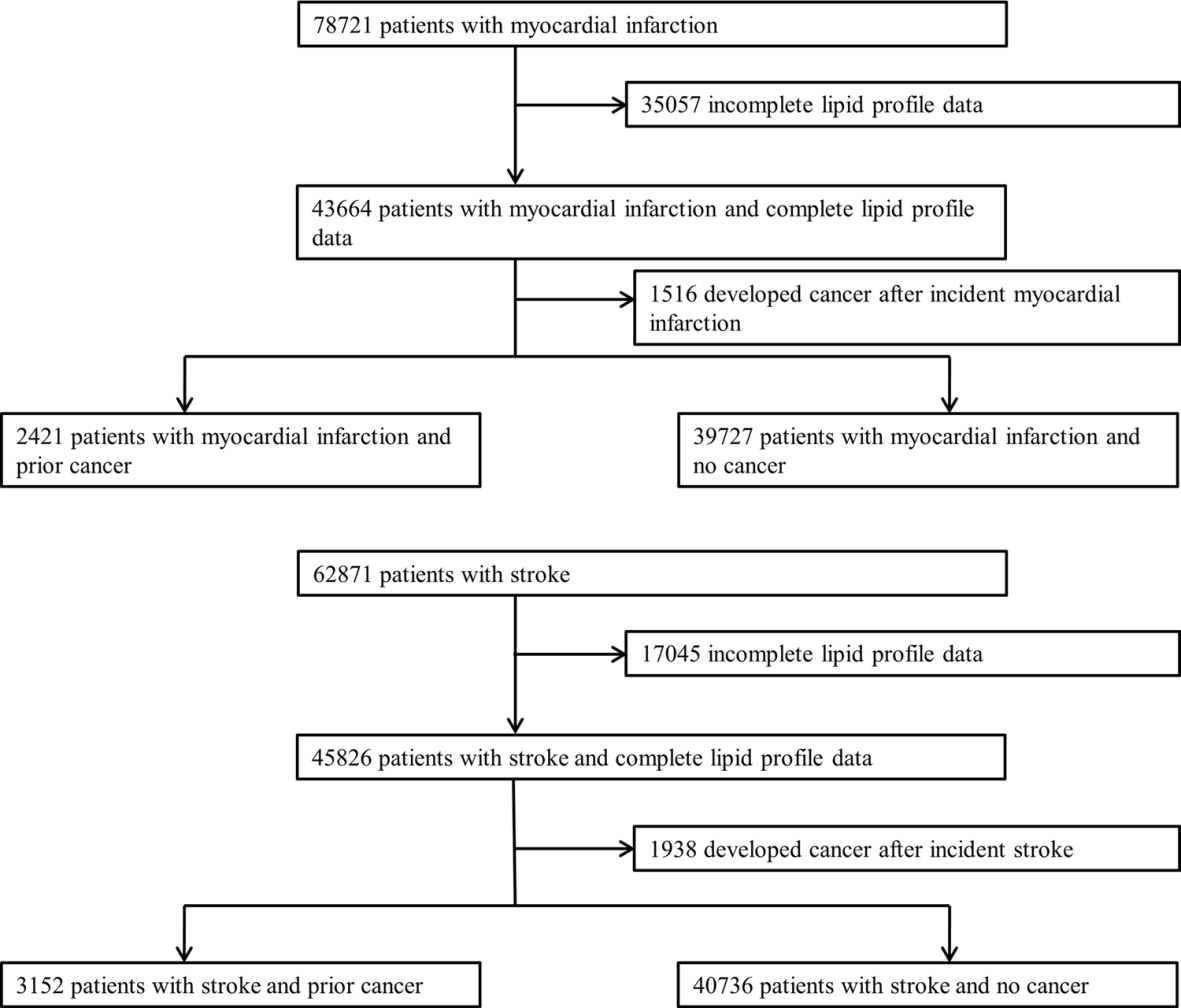
Flow chart of study patients. Study cohort profile for primary analysis.

We identified 78,721 individuals who incurred an incident MI between January 1, 2007 and 31 December, 2017. After excluding patients with incomplete cholesterol data and those who developed cancer after the incident MI, a total of 42,148 patients were analysed. There were 2421 patients who had prior cancer (prior cancer group) and 39,727 patients who did not have prior cancer (no cancer group). (Fig. 1) The median duration from cancer diagnosis to MI was 2251 days (6 years and 61 days). Baseline characteristics of the two groups at incident MI are presented in Table 1. The prior cancer group was older, and less likely to smoke, have hypertension, diabetes mellitus, renal impairment and hyperlipidaemia. The prior cancer group had a higher proportion of women and a lower median body mass index. The prior cancer group had fewer ST-segment elevation MIs and were less likely to undergo revascularization, and had higher rates of heart failure and cardiogenic shock. The median LDL-C concentration was significantly lower in the prior cancer group [2.43 mmol/L versus 3.10 mmol/L in the no cancer group; *p* < 0.001]. The prior cancer group also had significantly lower median triglyceride and total cholesterol concentrations and higher HDL-C concentration. At the point of discharge for the incident MI, the prior cancer group was less likely to be prescribed with guideline directed medical therapy including LLT.

**Table 1:** Demographic and clinical characteristics of patients at incident myocardial infarction.

	Prior cancer (n = 2421)	No cancer (n = 39,727)	*p* value
Age, median (IQR), years	74 (65–82)	62 (54–73)	< 0.001
Male sex, n (%)	1470 (60.7)	29,517 (74.3)	< 0.001
Body mass index, median (IQR), kg/m^2^	23.0 (20.4–25.9)	24.4 (22.0–27.3)	< 0.001
**Comorbidities, n (%)**			
Current/ex-smoker	972 (40.6)	20,758 (52.9)	< 0.001
Hypertension	1788 (73.9)	24,642 (62.1)	< 0.001
Hypertension receiving treatment	1440 (80.5)	18,789 (76.3)	< 0.001
Diabetes mellitus	975 (40.3)	14,207 (35.8)	< 0.001
Diabetes mellitus receiving treatment	772 (79.2)	11,121 (78.3)	0.509
Hyperlipidaemia	1370 (56.6)	20,831 (52.5)	< 0.001
Hyperlipidaemia receiving treatment	1035 (75.6)	14,501 (69.6)	< 0.001
Renal impairment*	692 (43.1)	6977 (27.5)	< 0.001
**MI characteristics, n (%)**			
STEMI	614 (27.2)	16,289 (42.1)	< 0.001
Previous MI	206 (8.5)	3242 (8.2)	0.547
Previous PCI	182 (7.6)	2911 (7.3)	0.697
Previous CABG	128 (5.3)	1339 (3.4)	< 0.001
Underwent revascularization	926 (38.3)	24,777 (62.4)	< 0.001
LVEF, median (IQR), %*	45 (35–57)	45 (35–55)	0.873
**Complications, n (%)**			
Cardiac arrest presentation	30 (1.2)	721 (1.8)	0.038
Heart failure	289 (11.9)	3396 (8.6)	< 0.001
Cardiogenic shock	87 (3.6)	1069 (2.7)	0.008
Stent thrombosis*	2 (0.1)	53 (0.2)	0.470
**Lipid profile, median (IQR), mmol/L**			
LDL-C	2.43 (1.80–3.24)	3.10 (2.30–3.94)	< 0.001
HDL-C	1.06 (0.83–1.31)	1.03 (0.86–1.25)	0.049
Triglyceride	1.21 (0.88–1.71)	1.36 (0.98–1.95)	< 0.001
Total cholesterol	4.16 (3.36–5.06)	4.84 (3.97–5.80)	< 0.001
**Medications at discharge, n (%)**			
Aspirin	1710 (78.1)	33,896 (90.4)	< 0.001
Beta-blocker	1690 (77.2)	31,478 (83.9)	< 0.001
ACE-I/ARB	1235 (56.4)	25,250 (67.3)	< 0.001
LLT	1960 (89.5)	35,822 (95.5)	< 0.001

*ACE-I* angiotensin-converting enzyme inhibitor, *ARB* angiotensin II receptor blocker, *CABG* coronary artery bypass grafting, *HDL-C* high density lipoprotein cholesterol, *IQR* interquartile range, *LDL-C* low density lipoprotein cholesterol, *LLT* lipid lowering therapy, *LVEF* left ventricular ejection fraction, *MI* myocardial infarction, *PCI* percutaneous coronary intervention, *STEMI* ST-segment elevation myocardial infarction.

Unknown values were excluded from the calculation of percentages.

*Data on renal impairment and stent thrombosis were available from 2012 onwards and data for LVEF were available from 2008 onwards.

Across the same timeframe, we identified 62,871 patients with an incident stroke. After excluding patients with incomplete cholesterol data and those who developed cancer after incident stroke, a total of 43,888 patients were analysed. There were 3152 patients in the prior cancer group and 40,736 patients in the no cancer group (Fig. 1). The median duration from cancer diagnosis to stroke was 2829 days (7 years and 274 days). Similar to the MI cohort, the prior cancer group was older and had a higher proportion of women (Table 2). The prior cancer group was less likely to smoke, have hypertension, diabetes mellitus, hyperlipidaemia, atrial fibrillation and previous ischaemic heart disease or stroke. The prior cancer group had higher NIHSS scores implying greater severity. The median LDL-C concentration was significantly lower in the prior cancer group [2.81 mmol/L versus 3.22 mmol/L in the no cancer group; *p* < 0.001]. The prior cancer group also had significantly lower triglyceride and total cholesterol concentrations, but higher HDL-C concentration than the no cancer group. The prior cancer group was less likely to receive thrombolysis. At the time of discharge for incident stroke, the prior cancer group was less likely to be prescribed guideline directed medical therapy including LLT.

**Table 2:** Demographic and clinical characteristics of patients at incident stroke.

	Prior cancer (n = 3152)	No cancer (n = 40,736)	*p* value
Age, median (IQR), years	75 (65–82)	66 (57–77)	< 0.001
Male sex, n (%)	1588 (50.4)	24,210 (59.4)	< 0.001
**Comorbidities, n (%)**			
Current/ex-smoker	995 (32.6)	15,783 (40.1)	< 0.001
Hypertension	2428 (87.7)	29,837 (78.7)	< 0.001
Diabetes mellitus	1080 (58.4)	14,286 (50.5)	< 0.001
Hyperlipidaemia	1835 (79.8)	21,699 (67.7)	< 0.001
Atrial fibrillation/flutter	716 (22.7)	6845 (16.8)	< 0.001
Ischaemic heart disease	699 (48.9)	7370 (34.0)	< 0.001
Previous TIA/stroke	510 (41.1)	6030 (30.7)	< 0.001
**Stroke characteristics, n (%)**			
Ischaemic	2996 (95.1)	37,590 (92.3)	< 0.001
Baseline NIHSS, median (IQR)*	5 (2–11)	4 (2–9)	< 0.001
Discharge NIHSS, median (IQR)	3 (1–8)	2 (1–6)	0.003
Thrombolysis*	147 (4.9)	2225 (5.9)	0.023
Endovascular therapy	6 (0.2)	85 (0.2)	0.828
**Lipid profile, median (IQR), mmol/L**			
LDL-C	2.81 (2.19–3.67)	3.22 (2.50–4.04)	< 0.001
HDL-C	1.14 (0.92–1.40)	1.10 (0.92–1.36)	0.002
Triglyceride	1.16 (0.88–1.60)	1.25 (0.90–1.77)	< 0.001
Total cholesterol	4.60 (3.80–5.53)	5.00 (4.20–5.91)	< 0.001
**Medications at discharge, n (%)**			
Anti-platelet*	1870 (69.7)	26,060 (76.4)	< 0.001
Anti-coagulation*	274 (10.2)	2983 (8.8)	0.010
LLT*	1629 (82.8)	20,996 (88.1)	< 0.001

*HDL-C* high density lipoprotein cholesterol, *IQR* interquartile range, *LDL-C* low density lipoprotein cholesterol, *LLT* lipid lowering therapy, *NIHSS* National Institutes of Health Stroke Scale, *TIA* transient ischaemic attack.

Unknown values were excluded from the calculation of percentages.

*Data on thrombolysis, anti-platelet, anti-coagulant and NIHSS scores were only available from 2008, 2009, 2010, and 2012 onwards respectively.

### LDL-C concentration at MI and strokes

**Figure 2:**
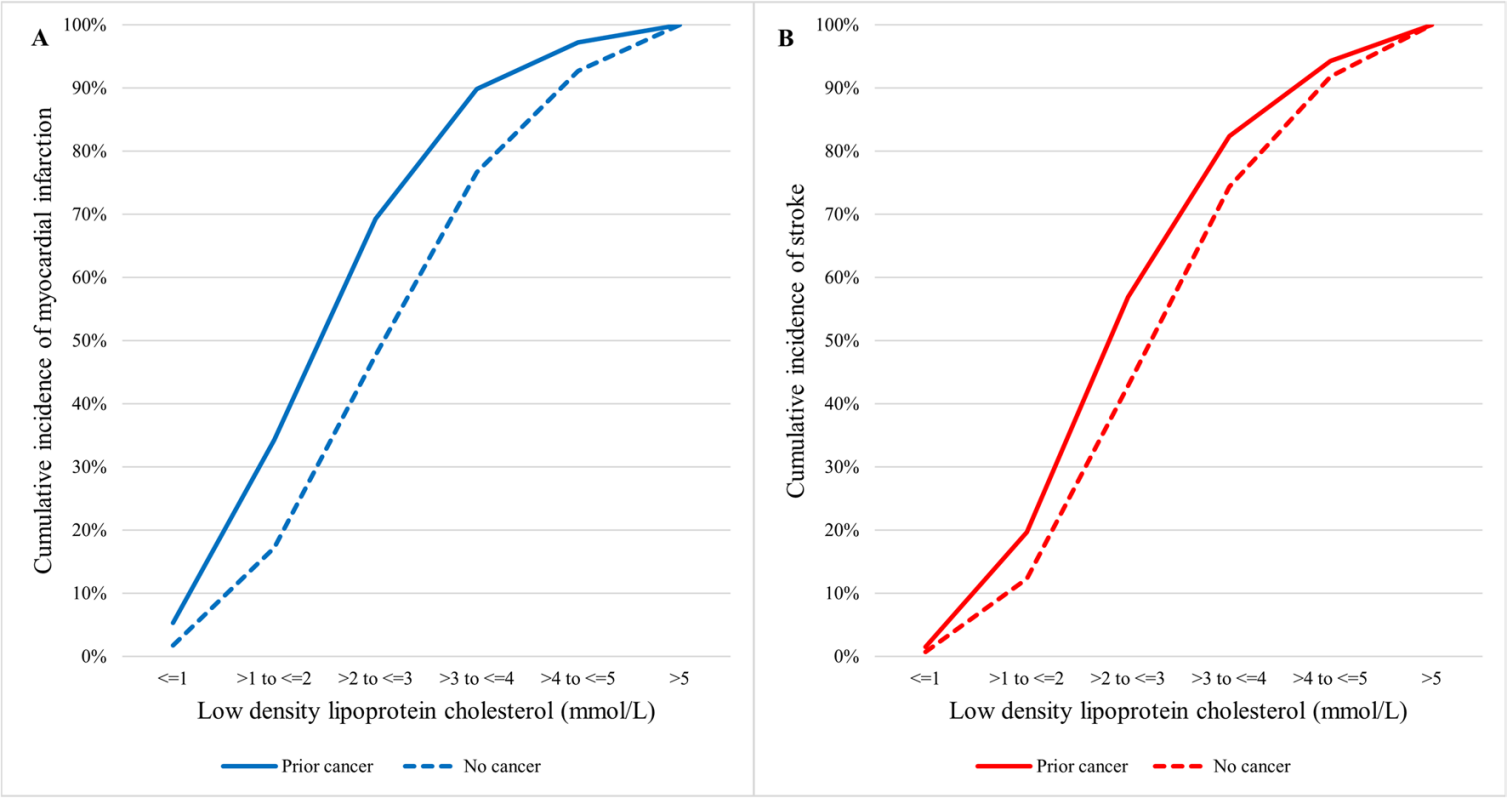
Cumulative incidence of myocardial infarction (**A**) and stroke (**B**) in relation to LDL-C by cancer status. The solid lines represent the prior cancer group whilst the dotted lines represent the no cancer group. LDL-C: low density lipoprotein cholesterol.

**Figure 3:**
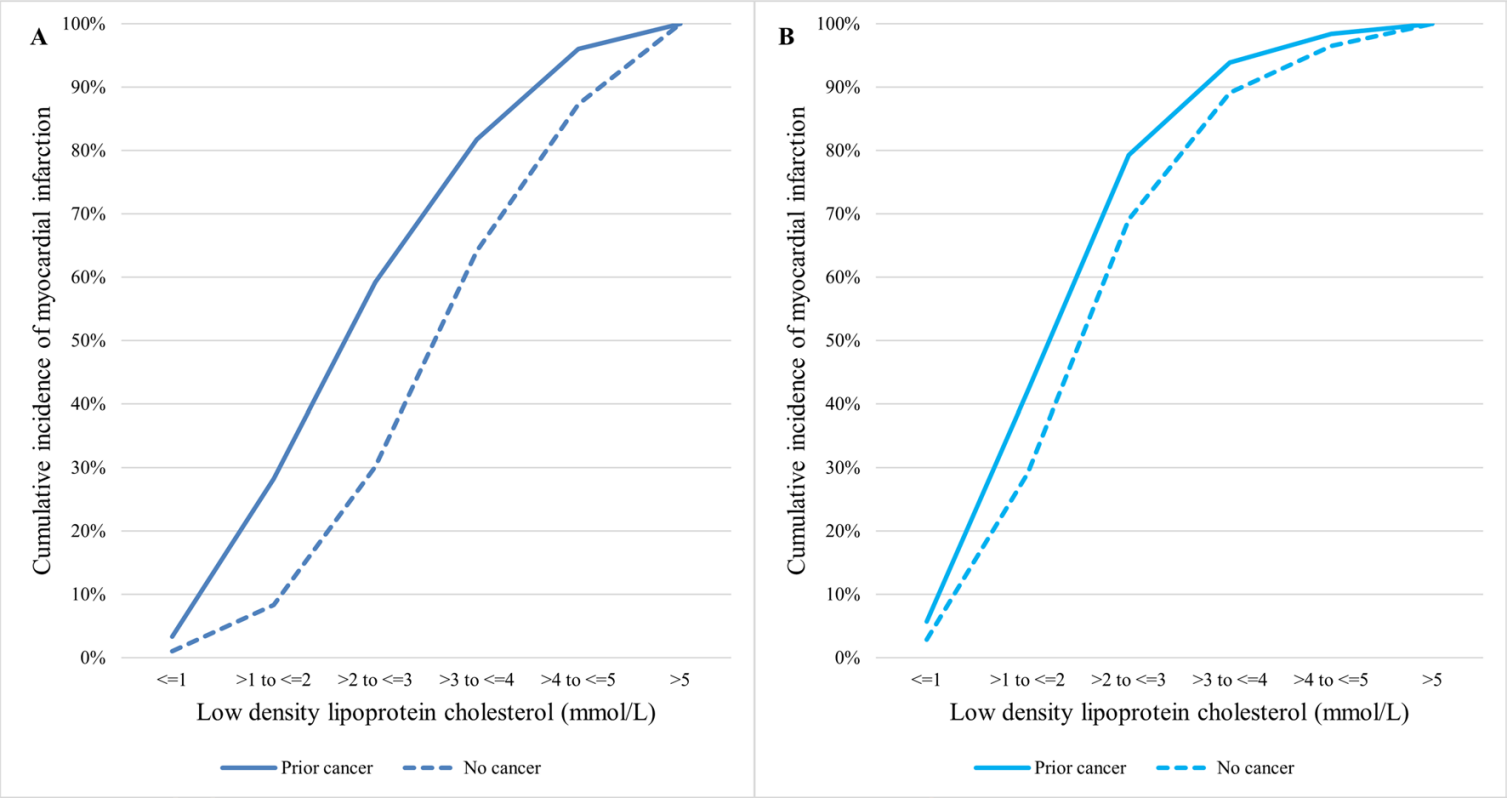
Cumulative incidence of myocardial infarction in patients not on prior LLT (**A**) and on prior LLT (**B**) in relation to LDL-C by cancer status. The solid lines represent the prior cancer group whilst the dotted lines represent the no cancer group. LDL-C: low density lipoprotein cholesterol; LLT: lipid lowering therapy.

Figure 2 shows the cumulative incidence of MI and stroke patients in relation to LDL-C concentration by prior cancer status. The proportion of MI and stroke patients in the prior cancer group was consistently higher at any given LDL-C concentration than the no cancer group. This observed difference in event rates between the prior cancer and no cancer groups was greater for incident MI than stroke events. Specifically for MI patients with history of hyperlipidemia, Fig. 3 shows the cumulative incidence of MI among prior cancer and no cancer groups stratified by whether patients were receiving LLT prior to the incident MI. In this subset of patients, there were 15,237 patients on prior LTT (1016 with prior cancer and 14,221 with no prior cancer) and 6507 patients not on prior LLT (329 with prior cancer and 6178 with no prior cancer). The use of prior LLT was associated with a significant attenuation in the difference in cumulative incidence of MI between prior cancer and no cancer groups across all LDL-C concentrations.

**Figure 4:**
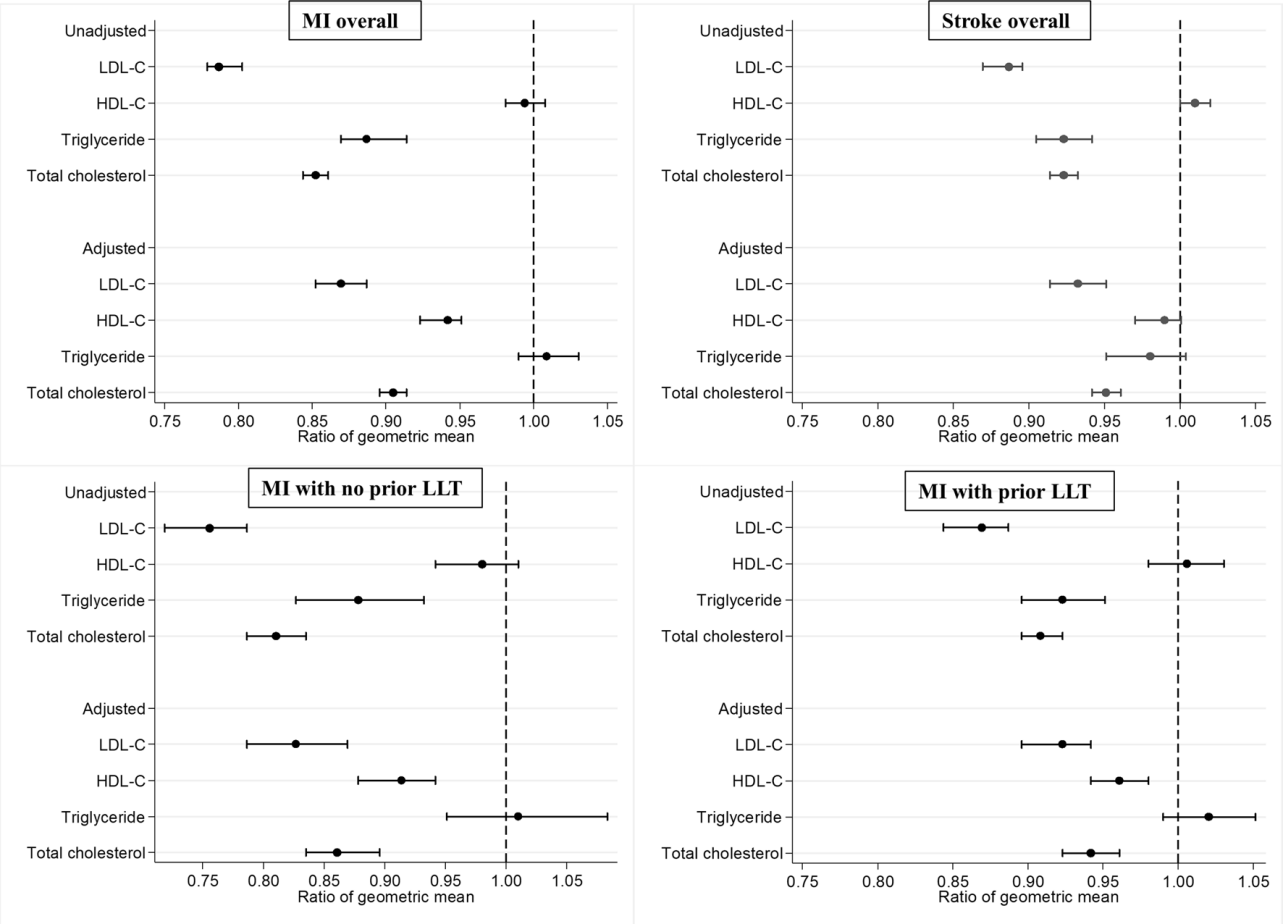
Ratio of geometric mean of lipid profile between patients in the prior cancer group and no cancer group (reference). Unadjusted and adjusted ratios of geometric mean of lipid profile divided into groups according to MI overall, stroke overall, MI with no prior LLT and MI with prior LLT. HDL-C: high density lipoprotein cholesterol; LDL-C: low density lipoprotein cholesterol; LLT: lipid lowering therapy; MI: myocardial infarction.

The geometric mean of LDL-C concentration at incident MI was 21% lower in the prior cancer group than the no cancer group [unadjusted ratio 0.79, 95% confidence interval (CI) 0.78–0.80] (Fig. 4). After adjustment for age, sex, body mass index, prior treatment for hypertension, prior treatment for diabetes mellitus, and prior treatment with LLT, the geometric mean of LDL-C concentration in the prior cancer group remained 13% lower than the no cancer group (adjusted ratio 0.87, 95% CI 0.85–0.89). There was no significant difference in triglyceride concentration, but the geometric means of both HDL-C and total cholesterol concentrations were significantly lower in the prior cancer group after adjustment (Fig. 4). The difference in geometric mean LDL-C concentration between the prior cancer and no cancer groups was significantly greater among patients who were not receiving LLT prior to the incident MI (adjusted ratio 0.83, 95% CI 0.79–0.87) than those who were receiving prior LLT (adjusted ratio 0.92, 95% CI 0.90–0.94) (Fig. 4). At incident stroke, the geometric mean of LDL-C concentration was 11% lower in the prior cancer group (unadjusted ratio 0.89, 95% CI 0.87–0.90), and remained 7% lower after adjustment (adjusted ratio 0.93, 95% CI 0.91–0.95). There was no significant difference in geometric mean triglyceride or HDL-C concentrations, but geometric mean total cholesterol was again significantly lower in the prior cancer group than the no cancer group (Fig. 4). Data on prior LLT use was not available in the Singapore stroke registry.

### LDL-C concentration among MI and stroke patients with prior cancer

The median LDL-C concentration at incident MI and stroke in relation to cancer characteristics are displayed in Table 3. There was no clinically significant difference in LDL-C according to sex, cancer staging and duration from cancer diagnosis. Across all cancer subtypes, the median LDL-C concentration was lower among patients with incident MI than stroke. Among those with MI, cancers involving the hepatobiliary system and pancreas had the lowest median LDL-C concentration. Among those with stroke, cancers involving the hepatobiliary system, pancreas, upper gastrointestinal tract and haematological malignancies had the lowest median LDL-C concentration. There was no significant difference in LDL-C concentration according to cancer treatment except in stroke patients who had radiotherapy.

**Table 3:** LDL-C at incident myocardial infarction or stroke in patients with prior cancer by clinical characteristics.

	Myocardial infarction		Stroke	
	LDL-C, median (IQR), mmol/L	*p* value	LDL-C, median (IQR), mmol/L	*p* value
**Sex**				
Male	2.49 (1.80–3.28)	0.144	2.76 (2.10–3.62)	0.002
Female	2.39 (1.74–3.19)		2.89 (2.22–3.70)	
Cancer TNM staging*				
I	2.49 (1.82–3.24)	0.282	2.80 (2.19–3.63)	0.624
II	2.41 (1.70–3.10)		2.70 (2.10–3.56)	
III	2.50 (1.77–3.31)		2.80 (2.16–3.70)	
IV	2.28 (1.60–3.40)		2.80 (2.09–3.82)	
**Duration from cancer diagnosis**^**a**^				
1st quartile	2.26 (1.53–3.00)	< 0.001	2.66 (2.02–3.49)	< 0.001
2nd quartile	2.56 (1.90–3.43)		2.90 (2.25–3.74)	
3rd quartile	2.50 (1.86–3.31)		2.90 (2.20–3.71)	
4th quartile	2.40 (1.83–3.19)		2.81 (2.20–3.65)	
**Cancer subtype**				
Head and neck	2.42 (1.80–3.40)	0.003	3.02 (2.33–4.10)	< 0.001
Upper gastrointestinal	2.23 (1.43–3.05)		2.59 (2.00–3.20)	
Hepatobiliary and pancreas	1.92 (1.42–2.80)		2.62 (1.82–3.17)	
Colorectal and anal	2.50 (1.85–3.33)		2.82 (2.10–3.60)	
Lung and pleura	2.33 (1.82–3.21)		2.86 (2.31–3.56)	
Thyroid	2.32 (1.73–3.34)		2.79 (2.19–3.60)	
Breast	2.48 (1.80–3.28)		2.90 (2.30–3.79)	
Gynaecological (Cervix, ovarian, vagina, endometrial)	2.32 (1.66–3.14)		2.98 (2.20–3.77)	
Urological (Kidney and bladder)	2.55 (1.95–3.31)		2.90 (2.13–3.63)	
Prostate	2.48 (1.89–3.20)		2.68 (2.10–3.55)	
Testicular and penile	2.60 (2.35–3.96)		3.00 (2.50–3.66)	
Haematological (Lymphoid and myeloid)	2.30 (1.61–3.10)		2.60 (2.00–3.41)	
Central nervous system (Brain, nervous and eye)	2.85 (1.93–3.97)		3.17 (2.59–3.70)	
Skin (Melanoma and non-melanoma)	2.50 (1.87–3.08)		2.67 (2.09–3.46)	
Others	2.50 (1.80–3.03)		2.75 (2.35–3.88)	
**Cancer treatment**^**b**^				
Surgery	2.45 (1.74–3.30)	0.187	2.74 (2.10–3.59)	0.747
No surgery	2.35 (1.70–3.14)		2.75 (2.10–3.60)	
Radiotherapy	2.40 (1.75–3.25)	0.689	2.90 (2.12–3.82)	0.012
No radiotherapy	2.40 (1.71–3.23)		2.70 (2.10–3.50)	
Chemotherapy	2.52 (1.80–3.29)	0.082	2.76 (2.12–3.63)	0.596
No chemotherapy	2.38 (1.70–3.20)		2.73 (2.10–3.60)	
Hormone therapy	2.40 (1.70–3.22)	0.691	2.78 (2.20–3.59)	0.618
No hormone therapy	2.40 (1.71–3.24)		2.73 (2.10–3.60)	
Biological therapy	2.50 (1.45–3.26)	0.580	2.54 (1.90–3.27)	0.173
No biological therapy	2.40 (1.71–3.24)		2.75 (2.10–3.60)	
**Clinical presentation**				
STEMI	2.75 (2.08–3.52)	< 0.001	–	–
NSTEMI	2.39 (1.76–3.16)			
Ischaemic stroke	–	–	2.80 (2.19–3.67)	0.923
Haemorrhagic stroke			2.90 (2.12–3.70)	

*IQR* interquartile range, *LDL-C* low density lipoprotein cholesterol, *NSTEMI* non ST-segment elevation myocardial infarction, *STEMI* ST-segment elevation myocardial infarction, *TNM* tumour node metastasis.

*Details on cancer staging are only available for cancer cases diagnosed from 2003 onwards.

^a^Quartile range differs between myocardial infarction and stroke cohorts. Myocardial infarction cohort quartiles: 0–715 days, 716–2251 days, 2252–4678 days, > 4678 days. Stroke cohort quartiles: 0–940 days, 941–2829 days, 2830–5526 days, > 5526 days.

^b^Details on cancer treatment are only available for cancer cases diagnosed from 2003 onwards; limited to treatment received within the first 6 months from cancer diagnosis.

### Post-MI and post-stroke mortality

Patients in the prior cancer group had a higher adjusted risk of all-cause mortality compared to patients in the no cancer group after the incident MI and stroke (Table 4). The association between prior cancer and post-MI mortality was significant at approximately 50% greater relative hazard of post-MI mortality with prior cancer, regardless if patients had prior LLT or no prior LLT before the MI. The association between prior cancer and post-stroke mortality was even more significant at 95% greater relative hazard of post-stroke mortality with prior cancer.

**Table 4:** Hazard ratio of all-cause mortality in myocardial infarction and stroke patients.

	Unadjusted HR (95% CI)	*p* value	Adjusted HR* (95% CI)	*p* value
**Post-MI mortality by prior cancer status among all MI patients**				
Prior cancer	2.71 (2.56–2.86)	< 0.001	1.48 (1.38–1.59)	< 0.001
No cancer	1.00 (reference)		1.00 (reference)	
**Post-MI mortality by prior cancer status among MI patients with history of hyperlipidemia**				
Prior cancer	2.20 (2.04–2.37)	< 0.001	1.34 (1.22–1.47)	< 0.001
No cancer	1.00 (reference)		1.00 (reference)	
**Post-MI mortality by prior LLT status among MI patients with history of hyperlipidemia**				
Prior LLT	1.60 (1.52–1.69)	< 0.001	1.05 (0.97–1.14)	0.245
No prior LLT	1.00 (reference)		1.00 (reference)	
**Post-MI mortality by prior cancer status and prior LLT status among MI patients with history of hyperlipidemia**				
Prior cancer, no prior LLT	3.47 (2.99–4.03)	< 0.001	1.59 (1.30–1.95)	< 0.001
Prior cancer, prior LLT	3.11 (2.83–3.43)	< 0.001	1.38 (1.21–1.58)	< 0.001
No cancer, prior LLT	1.66 (1.57–1.76)	< 0.001	1.08 (0.99–1.17)	0.080
No cancer, no prior LLT	1.00 (reference)		1.00 (reference)	
**Post-stroke mortality of cancer status among all stroke patients**				
Prior cancer	2.27 (2.16–2.38)	< 0.001	1.95 (1.52–2.52)	< 0.001
No cancer	1.00 (reference)		1.00 (reference)	

*CI* confidence interval, *HR* hazard ratio, *LLT* lipid lowering therapy, *MI* myocardial infarction.

*For MI: Adjusted for age at MI, sex, body mass index, smoking, prior treatment for hypertension, prior treatment for diabetes mellitus, LDL-C, left ventricular ejection fraction, ST-segment elevation MI, cardiac arrest, heart failure, and revascularisation.

*For stroke: Adjusted for age at stroke, sex, smoking, history of hypertension, history of diabetes mellitus, history of hyperlipidaemia, history of transient ischaemic attack or stroke, history of ischaemic heart disease, history or newly diagnosed atrial fibrillation or flutter, LDL-C, and baseline National Institutes of Health Stroke Scale score.

Although treatment with LLT prior to MI was not associated with lower post-MI mortality, when stratified by both prior cancer and prior LLT, patients with prior cancer not on LLT before the MI had the greatest risk of post-MI mortality (adjusted *p* < 0.001).

## Discussion

This study reports on the differences in plasma LDL-C concentrations at both incident MI and stroke among a nationwide cohort of patients with and without prior cancer. In comparing more than 40,000 patients with MI or stroke over a 10-year period, we showed that the adjusted geometric mean LDL-C concentration was 13% lower in patients with prior cancer than no cancer at incident MI and 7% lower in patients with prior cancer than no cancer at incident stroke. The difference in LDL-C between the prior cancer and no cancer groups was most pronounced in patients with MI who were not receiving LLT prior to the incident MI. Patients with prior cancer had a 48% higher post-MI mortality and a 95% higher post-stroke mortality, however the use of prior LLT was not associated with lower mortality post-MI.

There are several potential reasons why the mean LDL-C concentration at incident MI or stroke was lower among patients with prior cancer. Patients with cancer are inherently at a higher risk for adverse cardiovascular events^3–7^. This is despite a lack of a clear association between cancer and plasma lipid concentrations in prior limited case–control studies comparing patients with cancer against healthy controls^19,20^. Cancer is associated with increased inflammation, which plays a key role in atherosclerosis^21,22^. This is supported by the Canakinumab Anti-Inflammatory Thrombosis Outcomes Study (CANTOS), a large randomized trial which demonstrated that canakinumab, a human monoclonal antibody to the inflammatory cytokine interleukin-1β, reduced the risk of MI, stroke or cardiovascular death. Canakinumab achieved this benefit without any significant effect on plasma lipids including LDL-C^23^. Subgroup analyses further suggested that canakinumab reduced inflammatory markers and incident lung cancer^24^. This observation of an increased cardiovascular risk despite lower LDL-C concentration has also been previously described in patients with chronic inflammatory conditions such as rheumatoid arthritis, where low LDL-C concentration was associated with higher coronary artery calcium scores^25,26^. In addition, cancer therapies have been associated with cardiovascular toxicity in more than a third of all patients receiving treatment^8^. These included both overt adverse events such as heart failure and myocardial infarction, and early signs of toxicity such as abnormal cardiac biomarkers and subclinical left ventricular dysfunction. Recent studies have further demonstrated progression of atherosclerotic plaque volume after initiation of cancer therapies^27^.

The magnitude of difference in LDL-C between the prior cancer and no cancer groups was greater among patients with incident MI than among patients with incident stroke. In addition to shared atherosclerotic mechanisms, there are additional pathways for increased stroke risk in patients with cancer such as direct occlusion or haemorrhage from emboli or metastases, radiation therapy-induced vasculopathy, or the increased prevalence of atrial fibrillation^5^. We hypothesize that the presence of more potentially causative links between cancer and stroke, compared with cancer and MI, could account for the smaller difference in LDL-C concentration between patients with prior cancer and no cancer.

Despite having a lower LDL-C at incident MI, patients with prior cancer had a higher post-MI mortality rate. Patients with prior cancer not on LLT before incident MI had the highest post-MI mortality. This is consistent with previous studies demonstrating associations between statin therapy and lower mortality^28^. Although treatment with LLT prior to MI was not associated with lower post-MI mortality when stratified by both prior cancer and prior LLT, further prospective randomized trials are required to determine if LLT for patients with prior cancer can reduce MI events and potentially post-MI mortality.

Our findings have several clinical implications. First, our study highlights the need for improvement in cardiovascular primary prevention for an often neglected patient population. Patients with cancer have a higher prevalence of cardiovascular risk factors compared to patients without cancer^29^. These risk factors, including hyperlipidaemia, are often poorly controlled as healthcare workers tend to overlook promotion of ideal cardiovascular health behaviours to patients with cancer^30^. Physicians may be less inclined to prescribe LLT given the inherently lower LDL-C concentrations of patients with prior cancer, thereby denying these patients the preventative benefits of LLT against MI and stroke. Importantly, patients with cancer who develop MI or stroke have a higher post-MI or post-stroke mortality and accelerated cancer recurrence^31,32^. This highlights an important gap in knowledge and practice that can potentially reduce cardiovascular morbidity and improve outcomes in patients with cancer. Second, current guidelines on cholesterol management do not specifically address target lipid levels for patients with cancer^10,11^. The American College of Cardiology guidelines on management of blood cholesterol do mention atherosclerotic cardiovascular disease risk enhancers including inflammatory diseases such as rheumatoid arthritis, psoriasis, and infection with the human immunodeficiency virus^11^. We propose that a history of active or prior cancer should be included within this category of risk enhancers. We further propose that similar to rheumatoid arthritis, a position paper on lipid management in cancer is necessary to guide therapy and further research in this previously neglected patient group^33^. Given that the median LDL-C of patients with cancer who had a MI in our study was 2.4 mmol/L, our results suggest that a primary prevention target of a LDL-C concentration of less than 2.6 mmol/L may be inadequate in patients with prior cancer.

To the best of our knowledge, this is the first study to compare LDL-C concentrations between patients with prior cancer and no cancer at the point of incident MI or stroke. The Singapore disease registries are among the few universal registries that capture all-comers without the need for prior written informed consent, and hence there is low loss to follow-up and near complete capture of the entire national population^34–36^.

Our study has several limitations. First, details on prior LLT were only available in the MI registry and not the stroke registry. Second, additional treatment data on specific types of cancer therapy and duration of therapy administered were unavailable. Third, details on the intensity of statin therapy or the use of newer LLT agents such as ezetimibe or proprotein convertase subtilsin-kexin type 9 inhibitors, and follow-up of LDL-C concentrations were not available. Fourth, although there have been multiple changes in cholesterol guidelines throughout the years 2007 to 2017 affecting clinical practice, there appears to be minimal impact on the results of our study. Overall LDL-C concentrations at incident MI or stroke were highest during the years 2007 to 2009, and lowest in the years 2016 to 2017. The findings of lower LDL-C concentrations observed in the prior cancer group were consistent throughout the years. Finally, there was no data on inflammatory markers such as high sensitivity C-reactive protein or interleukin concentrations available for interpretation.

## Conclusion

In conclusion, prior cancer is associated with lower LDL-C at the time of incident MI or stroke. This difference in LDL-C concentration between prior cancer and no cancer groups is greater among patients with incident MI than among patients with incident stroke, and the between-group difference in LDL-C concentration is attenuated among patients initiated on LLT prior to the incident MI. Prior cancer was associated with poorer post-MI and post-stroke survival despite a lower LDL-C concentration at time of incident MI or stroke. Patients with prior cancer not on LLT before incident MI were at the greatest risk of post-MI mortality. Further research is needed to better define LDL-C treatment targets and the role and intensity of LLT in the prevention of MI and stroke among cancer survivors.

## Data Availability

The datasets generated during and analysed during the current study are available from the corresponding author on reasonable request.

## References

[1] Miller KD (2019). Cancer treatment and survivorship statistics, 2019. CA Cancer J. Clin..

[2] Coleman MP (2003). EUROCARE-3 summary: Cancer survival in Europe at the end of the 20th century. Ann. Oncol..

[3] Sturgeon KM (2019). A population-based study of cardiovascular disease mortality risk in US cancer patients. Eur. Heart J..

[4] Strongman H (2019). Medium and long-term risks of specific cardiovascular diseases in survivors of 20 adult cancers: A population-based cohort study using multiple linked UK electronic health records databases. Lancet.

[5] Zaorsky NG (2019). Stroke among cancer patients. Nat. Commun..

[6] Henson KE (2016). Cardiac mortality among 200 000 five-year survivors of cancer diagnosed at 15 to 39 years of age: The teenage and young adult cancer survivor study. Circulation.

[7] Kenzik KM (2018). New-onset cardiovascular morbidity in older adults with stage I to III colorectal cancer. J. Clin. Oncol..

[8] López-Sendón J (2020). Classification, prevalence, and outcomes of anticancer therapy-induced cardiotoxicity: The CARDIOTOX registry. Eur. Heart J..

[9] Libby P, Kobold S (2019). Inflammation: a common contributor to cancer, aging, and cardiovascular diseases: Expanding the concept of cardio-oncology. Cardiovasc. Res..

[10] Mach F (2020). 2019 ESC/EAS guidelines for the management of dyslipidaemias: Lipid modification to reduce cardiovascular risk. Eur. Heart J..

[11] Grundy SM (2019). 2018 AHA/ACC/AACVPR/AAPA/ABC/ACPM/ADA/AGS/APhA/ASPC/NLA/PCNA guideline on the management of blood cholesterol: A report of the American College of Cardiology/American Heart Association Task Force on Clinical Practice Guidelines. J. Am. Coll. Cardiol..

[12] Armenian SH (2017). Prevention and monitoring of cardiac dysfunction in survivors of adult cancers: American Society of Clinical Oncology Clinical Practice Guideline. J. Clin. Oncol..

[13] Curigliano G (2020). Management of cardiac disease in cancer patients throughout oncological treatment: ESMO consensus recommendations. Ann. Oncol..

[14] Koene RJ, Prizment AE, Blaes A, Konety SH (2016). Shared risk factors in cardiovascular disease and cancer. Circulation.

[15] National Reigistry of Diseases Office. https://www.nrdo.gov.sg (30 Aug 2020).

[16] Sim HW (2020). Beta-blockers and renin-angiotensin system inhibitors in acute myocardial infarction managed with inhospital coronary revascularization. Sci. Rep..

[17] Venketasubramanian N (2015). Countrywide stroke incidence, subtypes, management and outcome in a multiethnic Asian population: The Singapore Stroke Registry–methodology. Int. J. Stroke.

[18] Lee HP (1990). Monitoring cancer incidence and risk factors in Singapore. Ann. Acad. Med. Singap..

[19] Li D (2019). Comparative analysis of the serum proteome profiles of thyroid cancer: An initial focus on the lipid profile. Oncol. Lett..

[20] Siemianowicz K (2000). Serum total cholesterol and triglycerides levels in patients with lung cancer. Int. J. Mol. Med..

[21] Hansson GK (2005). Inflammation, atherosclerosis, and coronary artery disease. N. Engl. J. Med..

[22] Crusz SM, Balkwill FR (2015). Inflammation and cancer: Advances and new agents. Nat. Rev. Clin. Oncol..

[23] Ridker PM (2017). Antiinflammatory therapy with Canakinumab for atherosclerotic disease. N. Engl. J. Med..

[24] Ridker PM (2017). Effect of interleukin-1β inhibition with canakinumab on incident lung cancer in patients with atherosclerosis: exploratory results from a randomised, double-blind, placebo-controlled trial. Lancet.

[25] Myasoedova E (2011). Lipid paradox in rheumatoid arthritis: The impact of serum lipid measures and systemic inflammation on the risk of cardiovascular disease. Ann. Rheum. Dis..

[26] Giles JT (2019). Exploring the lipid paradox theory in rheumatoid arthritis: Associations of low circulating low-density lipoprotein concentration with subclinical coronary atherosclerosis. Arthritis Rheumatol..

[27] Drobni ZD (2020). Association between immune checkpoint inhibitors with cardiovascular events and atherosclerotic plaque. Circulation.

[28] Nielsen SF, Nordestgaard BG, Bojesen SE (2012). Statin use and reduced cancer-related mortality. N. Engl. J. Med..

[29] Weaver KE (2013). Cardiovascular risk factors among long-term survivors of breast, prostate, colorectal, and gynecologic cancers: A gap in survivorship care?. J. Cancer Surviv..

[30] Sabatino SA (2007). Provider counseling about health behaviors among cancer survivors in the United States. J. Clin. Oncol..

[31] Armenian SH (2016). Cardiovascular disease among survivors of adult-onset cancer: A community-based retrospective cohort study. J. Clin. Oncol..

[32] Koelwyn GJ (2020). Myocardial infarction accelerates breast cancer via innate immune reprogramming. Nat. Med..

[33] Hollan I (2020). Lipid management in rheumatoid arthritis: A position paper of the working group on cardiovascular pharmacotherapy of the European society of cardiology. Eur. Heart J. Cardiovasc. Pharmacother..

[34] Wu CK (2018). The Taiwan heart registries: Its influence on cardiovascular patient care. J. Am. Coll. Cardiol..

[35] Taylor J (2009). SWEDEHEART: Sweden’s new online cardiac registry, the first of its kind. Eur. Heart J..

[36] Copenhagen Healthtech Cluster. Danish Heart Registry. https://www.danishhealthdata.com/find-health-data/Dansk-Hjerteregister (30 Aug 2020).

